# Prefusion RSV F Immunization Elicits Th2-Mediated Lung Pathology in Mice When Formulated With a Th2 (but Not a Th1/Th2-Balanced) Adjuvant Despite Complete Viral Protection

**DOI:** 10.3389/fimmu.2020.01673

**Published:** 2020-07-29

**Authors:** Katherine M. Eichinger, Jessica L. Kosanovich, Sonal V. Gidwani, Aaron Zomback, Madeline A. Lipp, Timothy N. Perkins, Tim D. Oury, Nikolai Petrovsky, Christopher P. Marshall, Mark A. Yondola, Kerry M. Empey

**Affiliations:** ^1^Department of Pharmacy and Therapeutics, University of Pittsburgh School of Pharmacy, University of Pittsburgh, Pittsburgh, PA, United States; ^2^Center for Clinical Pharmaceutical Sciences, University of Pittsburgh School of Pharmacy, University of Pittsburgh, Pittsburgh, PA, United States; ^3^Department of Medicine, Division of Internal Medicine, University of Pittsburgh School of Medicine, Pittsburgh, PA, United States; ^4^Calder Biosciences, New York City, NY, United States; ^5^Department of Pathology, University of Pittsburgh School of Medicine, Pittsburgh, PA, United States; ^6^Vaxine Pty Ltd., Bedford Park, SA, Australia; ^7^College of Medicine and Public Health, Flinders University, Bedford Park, SA, Australia; ^8^Department of Immunology, University of Pittsburgh School of Medicine, University of Pittsburgh, Pittsburgh, PA, United States

**Keywords:** RSV, immunization, prefusion, infection, Th1/Th2-immunity, adjuvant

## Abstract

Respiratory syncytial virus (RSV) remains the most common cause of lower respiratory tract infections in children worldwide. Development of a vaccine has been hindered by the risk of developing enhanced respiratory disease (ERD) upon natural exposure to the virus. Generation of higher quality neutralizing antibodies with stabilized pre-fusion F protein antigens has been proposed as a strategy to prevent ERD. We sought to test whether there was evidence of ERD in naïve BALB/c mice immunized with an unadjuvanted, stabilized pre-fusion F protein, and challenged with RSV line 19. We further sought to determine the extent to which formulation with a Th2-biased (alum) or a more Th1/Th2-balanced (Advax-SM) adjuvant influenced cellular responses and lung pathology. When exposed to RSV, mice immunized with pre-fusion F protein alone (PreF) exhibited increased airway eosinophilia and mucus accumulation. This was further exacerbated by formulation of PreF with Alum (aluminum hydroxide). Conversely, formulation of PreF with a Th1/Th2-balanced adjuvant, Advax-SM, not only suppressed RSV viral replication, but also inhibited airway eosinophilia and mucus accumulation. This was associated with lower numbers of lung innate lymphocyte cells (ILC2s) and CD4+ T cells producing IL-5+ or IL-13+ and increased IFNγ+ CD4+ and CD8+ T cells, in addition to RSV F-specific CD8+ T cells. These data suggest that in the absence of preimmunity, stabilized PreF antigens may still be associated with aberrant Th2 responses that induce lung pathology in response to RSV infection, and can be prevented by formulation with more Th1/Th2-balanced adjuvants that enhance CD4+ and CD8+ IFNγ+ T cell responses. This may support the use of stabilized PreF antigens with Th1/Th2-balanced adjuvants like, Advax-SM, as safer alternatives to alum in RSV vaccine candidates.

## Introduction

Respiratory syncytial virus (RSV) is the most common cause of lower respiratory tract infections (LRTI) in children worldwide with nearly every child infected by 2 years of age (1–3). In children >5 years of age, RSV causes an estimated 33 million acute LRTI annually, with over 3 million episodes requiring hospitalization (4). In 2017, the global cost estimate for inpatient and outpatient RSV LRTI management in young children (<5 years of age) was ~4.8 billion euros (equivalent to ~$5.2 billion USD) (5). In addition to young children, RSV is a common cause of severe respiratory disease in the elderly and those who are immunocompromised (6–8). Given that severe RSV disease affects ages spanning infancy to geriatrics, it is clear that natural RSV infection does not induce long-lasting immunity and individuals are re-infected throughout their lives (9, 10). Thus, RSV immunization has the potential to boost RSV immunity and alleviate the morbidity associated with repeated RSV infections across all age groups. However, despite the immense economic and healthcare burden posed by RSV infection, there is currently no licensed RSV vaccine.

The discovery and stabilization of the prefusion conformation of the RSV F protein (PreF) re-ignited hopes for an RSV vaccine due to its ability to elicit potent neutralizing antibodies (11). A number of studies have demonstrated the protective potential of high levels of RSV neutralizing antibody and as such, boosting serum neutralizing antibody levels has been an important objective of RSV vaccine research (12, 13). The incorporation of adjuvants into vaccine formulations can enhance the vaccine's effect as well as reduce antigen concentrations and the number of immunizations required for a protective effect (14). Differential stimulation of various pattern recognition receptors can further shift the immune response toward vaccine antigens to promote Th1- or Th2-type immune responses. Based on the known Th2-bias associated with early RSV infections, it is imperative to understand the extent to which preventative RSV vaccine adjuvants shift the Th1/Th2 balance. In use since the 1930's, aluminum salts have long been recognized for their ability to enhance immunogenicity and boost antibody production (15). Alum adjuvant was used in the formalin-inactivated RSV (FI-RSV) trials of the 1960's that produced enhanced respiratory disease (ERD) requiring hospitalization upon natural RSV exposure in 80% of vaccinees (16). Subsequent investigations into the cause of FI-RSV-induced ERD have suggested that alum exacerbated the T helper type 2 (Th2) pathology associated with ERD (17). Other studies have disputed alum's role and have instead suggested that formalin inactivation of RSV resulted in poor neutralizing antibody development and immune complex deposition (18, 19). Reports have also demonstrated that formalin-inactivation of RSV resulted in post-fusion F (PostF) protein being the predominant protein presented on the surface of the virion (20). The implication of this data is that PreF is more representative of live RSV and therefore, may be less likely than PostF subunit vaccines to induce pathology. Moreover, in a naïve cotton rat model, both PreF and PostF immunization elicited protective RSV immunity without inducing alveolitis when paired with the Th1-skewing toll-like receptor 4 agonist (TLR4), glucopyranosyl lipid A (GLA) (21). These results suggest that more Th1-biased adjuvants may provide a safe alternative to alum in models of PreF immunization. Importantly, Th1-skewing adjuvants, including TLR9 agonists, have recently been FDA-approved for use in other vaccine systems, like Hepatitis B (22). Advax-SM is an adjuvant comprised of delta inulin polysaccharide formulated with the TLR9 agonist, CpG oligodeoxynucleotides (CpG ODN). The Advax-SM adjuvant system has demonstrated greater Th1-skewing properties when formulated with live RSV immunization (23) and ameliorated Th2-related airway eosinophilia in a model of immunization against severe acute respiratory syndrome (SARS)-associated coronavirus (24).

In the immunization studies presented here, we conducted a detailed evaluation of the protective capacity of RSV PreF antigen alone or combined with the Th1/Th2-balanced adjuvant, Advax-SM (PreF/Advax-SM), or the Th2-skewing adjuvant, Alum (PreF/Alum/aluminum hydroxide) in naïve BALB/c mice. These studies evaluated immunogenicity, efficacy, innate and adaptive immune responses, and safety at acute and convalescent time points following RSV challenge. Despite PreF/Alum immunization generating higher neutralizing antibody titers, both PreF/Advax-SM and PreF/Alum had undetectable viral replication, while PreF alone lacked the immunogenicity to fully protect from RSV infection in naïve animals. PreF/Advax-SM induced more balanced Th1/Th2 immunity characterized by the generation of neutralizing antibody, a mean PreF-specific IgG2a/IgG1 ratio >2, RSV F-specific and cytotoxic CD8+ T cells, and Th1 CD4+ T cells. Importantly, PreF/Advax-SM immunization protected from increased inflammation and mucus production, even when compared to PBS controls. In contrast, PreF alone and PreF/Alum generated robust Th2 immunity evidenced by PreF-specific IgG2a/IgG1 ratios <1 and increased IL-5+ and IL-13+ CD4+ T cells. Interestingly, despite undetectable viral replication at 4dpi, PreF/Alum immunization induced large populations of type 2 innate lymphoid cells (ILC2) in the lung producing the Th2-type cytokines, IL-5 and IL-13. Combined, the Th2 immunity of PreF/Alum was consistent with enhanced inflammation featuring airway eosinophils and increased mucus production. Overall, our observations have significant implications for the RSV vaccine field demonstrating that higher neutralizing antibody titers, while protective, are not implicitly tied to RSV PreF vaccine safety and more Th1/Th2-balanced adjuvants may generate protective responses while eliciting a desirable safety profile in naïve mice.

## Materials and Methods

### Mice, Immunization, and RSV Virus

Animal studies were carried out in accordance with the University of Pittsburgh's IACUC guidelines for the use and care of laboratory animals. Seven to eight week old Balb/cJ female mice were purchased from The Jackson Laboratory (Bar Harbor, ME). Female mice were immunized via intramuscular (i.m.) injection with 50 mcl of vehicle (Naïve and PBS), stabilized RSV prefusion protein (PreF; 10 mcg; Calder Biosciences) alone, or formulated with Advax-SM™ (PreF/Advax-SM; 1 mg/mouse; Vaxine Pty Ltd, Bedford Park, Australia) or Alum (PreF/Alum; 2 mg/mL). Specifically, Alhydrogel adjuvant 2%, an aluminum hydroxide wet gel suspension from InvivoGen, was used in these studies. Advax-SM is composed of microparticles of polyfructofuranosyl-_D_-glucose (delta inulin) combined with CpG55.2-ODN (5′ATCGACTCTCGAGCGTTCTC-3′), which was synthesized by GeneDesign (Osaka, Japan). The immunized mice were boosted 3 weeks later with their respective vaccine formulations. At 9 weeks post-prime, mice were challenged intranasally (i.n) with RSV L19 (5 × 10^5^ pfu/gm) and culled at 4 or 8 days post-infection (dpi) using 100% isoflurane and cervical dislocation. RSV L19 was propagated and viral titers quantified as previously described (25). All animal studies were approved by the University of Pittsburgh Institutional Animal Care and Use Committee; protocol #20047209.

### Cell Preparation, Cytokine Analysis, and Flow Cytometry

Bronchoalveolar lavage (BAL) was collected through intratracheal instillation of HBSS + EDTA. BAL samples were centrifuged and the soluble fraction stored at −80°C for cytokine analysis and the cellular fraction analyzed via flow cytometry. Cytokine concentrations were determined using the Bio-Plex Pro™ Mouse Cytokine 23-plex Assay (BioRad, CA), per manufacturer's protocol. The right lung was harvested and enzyme-digested into a single cell suspension for flow cytometry, as described previously (26, 27). Where indicated, BAL cells and lung homogenate were stimulated *ex-vivo* for intracellular cytokine detection. Briefly, live cells from BAL and lung homogenate were enumerated using a hemacytometer and trypan blue. For intracellular cytokine staining of lung homogenate or BAL, 1 million cells in duplicate (singular for BAL) were plated in a CD3-coated (5 mcg/mL, Biolegend) 96-well flat-bottomed tissue culture plate in 200 mcl of 10% RPMI supplemented with CD28 (2 mcg/mL) and incubated at 37°C overnight. After overnight stimulation with CD3/CD28, lung homogenate underwent a secondary stimulation with PMA (1:1,000), ionomycin (1:1,000), and Brefeldin A (1:1,000) for 2 h prior to T cell surface and intracellular flow staining. To obtain intracellular ILC2 cytokine staining, lung homogenate (3 million cells) was plated in 24-well tissue culture plates and stimulated with PMA (30 ng/mL), ionomycin (500 ng/mL), and Brefeldin A (1:1,000) in 10% RPMI at 37°C for 3 h prior to surface and intracellular flow staining.

BAL cells were surface stained with combinations of the following (clone): Molecular Probes LIVE/DEAD Fixable Blue, CD16/32 (2.4G2), Siglec-F (E50-2440), F4/80 (T45-2342), CD11b (M1/70), Ly6G (1A8), CD4 (GK1.5), CD8α (53-6.7), CD44 (IM7) (BD Biosciences, CA), CD11c (N418), CD19 (6D5), and TCR_β_(H57-597) (Biolegend, CA). Lung homogenate was surface stained with combinations of the following antibodies: CD16/32 (2.4G2), Lineage cocktail, CD45 (30-F11), ST2 (DIH9), IL-7Rα (A7R32), CD19 (6D5), TCR_β_(H57-597) (Biolegend), CD4 (GK1.5), and CD8α (53-6.7) (BD Bioscience). Following surface staining, cells were fixed and permeabilized for intracellular staining with BD CytoFix/CytoPerm™ Solution Kit (BD Biosciences) according to the manufacturer's protocol. Intracellular cytokines were stained with a combination of the following: CD206 (C068C2), IL-5 (TRFK5), IFNγ (XMG1.2), Granzyme B (QA16A02) (Biolegend), and IL-13 (ebio13A) (ThermoFisher Scientific, MA). Where indicated and prior to surface staining, BAL samples were incubated with RSV A Strain F-protein _85−93_ MHC I pentamer (H-2kd KYKNAVTEL; Proimmune, FL) to identify RSV F_85−93_ -specific CD8^+^ T cells. Samples were run on a BD LSRFortessa managed by the United Flow Core of the University of Pittsburgh. Data was analyzed using FlowJo V10 software (FLOWJO, LLC, OR). Cell populations were defined as follows: eosinophils (Siglec F+/ F4/80+/ CD206lo/-/ CD11b+); neutrophils (Siglec F-/ CD11bHI/ Ly6G+/ CD11c-/lo); monocytes (Siglec F–/ F4/80+/ CD11c+/ CD11b+); ILC2s (Lin–/ CD45+/ ST2+/ IL-7Rα+); T cells (± CD19-/ TCR_β_+/ CD4+ or CD8+). A LPS-treated negative control was used to set the gate for RSV A Strain F-protein _85−93_ MHC I pentamer+ CD8+ T cells.

### Neutralizing Antibody - Renilla Luciferase Rsv Reporter Assay

Pre-challenge serum was collected via submandibular bleed 2–3 days prior to RSV challenge and separated using Gel-Z Serum Separator Tubes (Sarstedt, Germany). Serum was stored at −80°C until heat inactivation (56°C for 30 min) and neutralizing antibody titers were performed. Serial dilutions of heat inactivated serum (50 mcL in phenol-free MEM supplemented with 5% FBS and Pen/Strep, Invitrogen) were incubated for 2 h in a 37°C CO_2_ incubator in a 96-well plate format with 100 pfu/well Line 19 RSV-Renilla Luciferase virus (provided by Martin Moore) in 50 mcL phenol free MEM medium as above. After 2 h, Hep-2 cells were trypsinized and a total of 2.5 × 10^4^ cells were added per well in 25 mcL of phenol free MEM with FBS and antibiotics as above. Cells were incubated for a total of 64–66 h at 37°C, 5% CO_2_ and luciferase readout was then obtained using the Renilla-glo luciferase kit (Promega) according to the manufacturer's instructions. Luciferase activity (luminescence) was measured using a Novostar plate reader after a 15 min incubation at 25°C. All plates were run in duplicate and averaged.

### Pre-F Specific IgG Subtype Assay

Co-star 96-well, high binding ELISA plates were coated with RSV PreF at a concentration of 5 mcg/ml overnight at 4°C. Each plate included standards of either mouse IgG1 or IgG2a (Invitrogen) at 10 and 2 mcg/mL in a 2-fold dilution series for intra-plate quantification of signal on uncoated wells. Plates were then washed with PBS, and blocked for 1 h at 37°C with 1% BSA in PBS. Heat-inactivated serum samples were diluted 1:500 in 1% BSA in PBS for the first well, and then 3-fold serially diluted a total of 3 times. Serum was incubated on the plates for 1 h at 25°C, followed by three washes with PBS 0.05% Tween-20, and secondary antibody incubation with anti-IgG1 or anti-IgG2a (isotype specific, BD Pharmingen), respectively at a 1:10,000 dilution for 30 min at 25°C in 1% BSA. 1-step TMB (Thermo Scientific) was used to develop the plates and the reaction was quenched by the addition of 4 N H_2_SO_4_. Plates were read at 450 nm in a Novostar plate reader. Data analysis was performed in Excel and data points were interpolated from the linear region of the standards on each individual plate. Samples were run in duplicate and the data presented represents the average values from both runs.

### Histology

Left lungs were gravity filled with 10% formalin at 4 and 8 days post RSV challenge, as previously described (28). The McGowan Institute for Regenerative Medicine (University of Pittsburgh, PA) stained and processed the preserved lungs. Periodic Acid-Schiff (PAS) stained lungs were assessed by two pathologists blinded to treatment groups to quantify airway mucus production, according to previously published methods (26). Briefly, a score of 0–4 was given to all airways (average 50) with the following scale: 0 = no PAS+ cells; 1 = 1–25% PAS+ cells; 2 = 26–50% PAS+ cells; 3 = 51–75% PAS+ cells; 4 = 76–100% PAS+ cells. Scores were averaged and the total percentage of PAS+ airways were graphed along with a more detailed breakdown of the proportion of each severity score (0–4) (# of airways of individual severity scores/total airways scored). Standard hematoxylin and eosin (H&E) staining was performed on lung sections and scored by two pathologists (Oury and Perkins) blinded to treatment groups, according to previously described methods (29). In short, each field (average 28 fields) in the lung was observed with a light microscope (x200 magnification) and scoring was based on the percentage of lung tissue affected according to the following scale: 0 = no inflammation, 1 = up to 25%, 2 = 25–50%, 3 = 50–75%, and 4 = 75–100%. Scores were averaged and reported as a proportion of the sum of scores divided by the total number of fields counted.

### Statistical Analysis

Statistics were performed with GraphPad Prism 8 software (GraphPad Software, La Jolla, CA). Results in the figures are displayed as the mean ± SEM. Neutralizing antibody data was analyzed by nonlinear regression to obtain IC_50_ values, which were compared between immunization groups using ANOVA with a Tukey's post-test, with PBS serving as the control. Statistical significance was determined between each immunization cohort using ANOVA with Tukey's multiple comparison test between all groups (Note: Naive animals were not included in analysis but are graphed for reference). Comparisons of the inflammatory and % PAS+ scores within immunization groups over time were made using *t*-tests corrected for multiple comparisons with the Holm-Sidak method (α = 0.05). *P*-values < 0.05 were considered significant. Data is representative of 2 separate experiments.

## Results

### The Th2-Biased Humoral Immunity of PreF/Alum and the Greater Th1-Skewed Humoral Immunity of PreF/Advax-SM Protect Against RSV Replication

**Figure 1:**
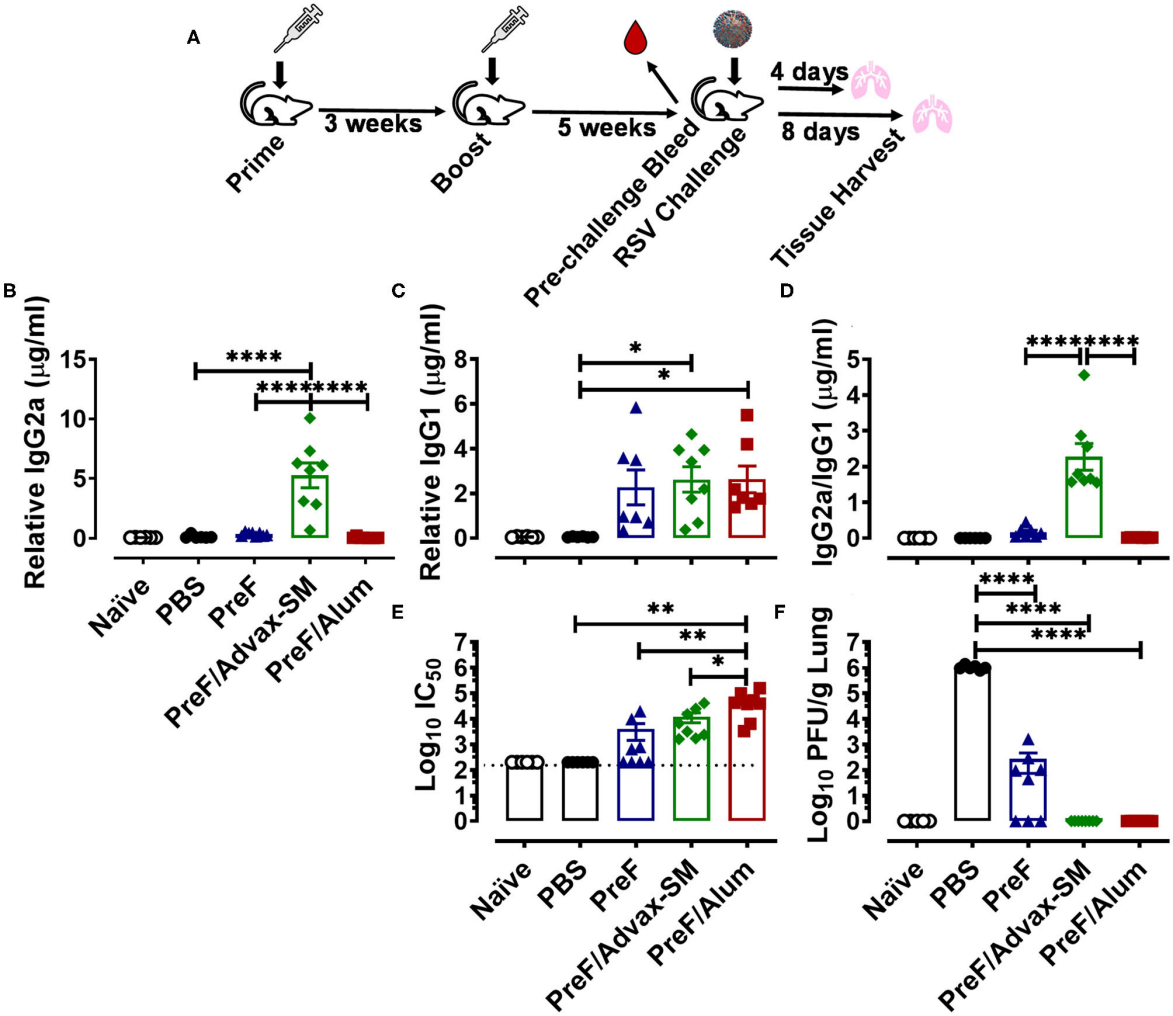
The Th2-biased humoral immunity of PreF/Alum and the Th1 skewed-humoral immunity of PreF/Advax-SM protect from RSV challenge. At 7–8 weeks of age, female adult BALB/cJ mice were immunized with phosphate buffered saline vehicle control (naïve & PBS), prefusion RSV F protein alone (PreF), or PreF formulated with Advax-SM (PreF/Advax-SM) or Alum (PreF/Alum) as depicted in **(A)**. Six weeks post-boost, pre-challenge serum was collected and mice were subsequently intranasally challenged with vehicle (naïve) or 5 × 10^5^ PFU/gram of RSV Line 19. Animals were culled for sample collection at 4 or 8 days post infection (dpi) **(A)**. Pre-challenge serum was analyzed for PreF-specific IgG2a **(B)**, IgG1 **(C)**, IgG2a to IgG1 ratios **(D)** and neutralizing antibody titers **(E)**. At 4 dpi, left lungs were harvested and virus quantified using standard H&E plaque assays **(F)**. Data are represented as mean ± SEM (*n* = 6-8 mice per group);*p < 0.05, **p < 0.01, and ****p < 0.0001. Due to a lack of pre-existing PreF-specific antibody, PBS was not included in the analysis in **(D)**.

The objective of this study was to determine the capacity of RSV pre-fusion (PreF) vaccines formulated with different adjuvants to generate neutralizing antibody, prevent virus replication, and protect from pulmonary pathology following RSV challenge. To that end, 8 week old BALB/cJ mice were immunized twice with PBS (vehicle control), PreF alone, or PreF formulated with the Th1/Th2-balanced adjuvant, Advax-SM (PreF/Advax-SM), or the T helper type 2 (Th2)-skewing adjuvant, aluminum hydroxide (PreF/Alum). Six weeks after their final immunization, sera were collected from mice in each group prior to viral challenge with RSV line 19; a control group of PBS-immunized mice received vehicle only (Naïve) (Figure 1A).

A comparison of PreF-specific IgG2a and IgG1 in pre-challenge sera provided initial evidence supporting the roles of Advax-SM as a more Th1-biased adjuvant and Alum as a Th2-polarizing adjuvant; production of IgG2a and IgG1 subclasses are reflective of their respective Th1 and Th2 biased immune responses in mice (30). PreF/Advax-SM immunization produced higher titers of IgG2a than all immunization groups tested (Figure 1B) and greater IgG1 compared to PBS (Figure 1C). The mean IgG2a/IgG1 ratio of PreF/Advax-SM mice was > 2 and significantly higher than PreF or PreF/Alum animals, indicating a more Th1-skewed humoral response (Figure 1D). In contrast, PreF/Alum immunization produced negligible titers of IgG2a (Figure 1B) and instead elicited increased titers of IgG1 (Figure 1C), with a resulting IgG2a/IgG1 ratio <1 (Figure 1D). Similar to PreF/Alum, PreF alone elicited an IgG2a/IgG1 ratio <1, indicating Th2-dominant antibody responses in both groups.

To assess differences in protective antibody responses between immunization groups, neutralizing antibody titers were measured in pre-challenge sera and RSV lung titers were quantified at 4 days post-infection (dpi). Mice immunized with PreF/Alum generated greater neutralizing antibody titers than all other immunization groups (Figure 1E). Despite lower neutralizing antibody titers in mice immunized with PreF/Advax-SM, as compared to PreF/Alum, both immunization groups had undetectable RSV in their lungs at 4 dpi (Figure 1F). In contrast, PreF alone generated measurable neutralizing antibody titers in 50% (*n* = 4/8) of the animals and only 38% (*n* = 3/8) had undetectable RSV lung titers (Figure 1F). All immunized mice had lower viral lung titers when compared to PBS controls following RSV challenge, however, only mice immunized with PreF/Alum or PreF/Advax-SM achieved full viral protection at 4 dpi.

### PreF and PreF/Alum Immunization Elicited Enhanced Pulmonary Inflammation Characterized by Robust Airway Eosinophil Recruitment

**Figure 2:**
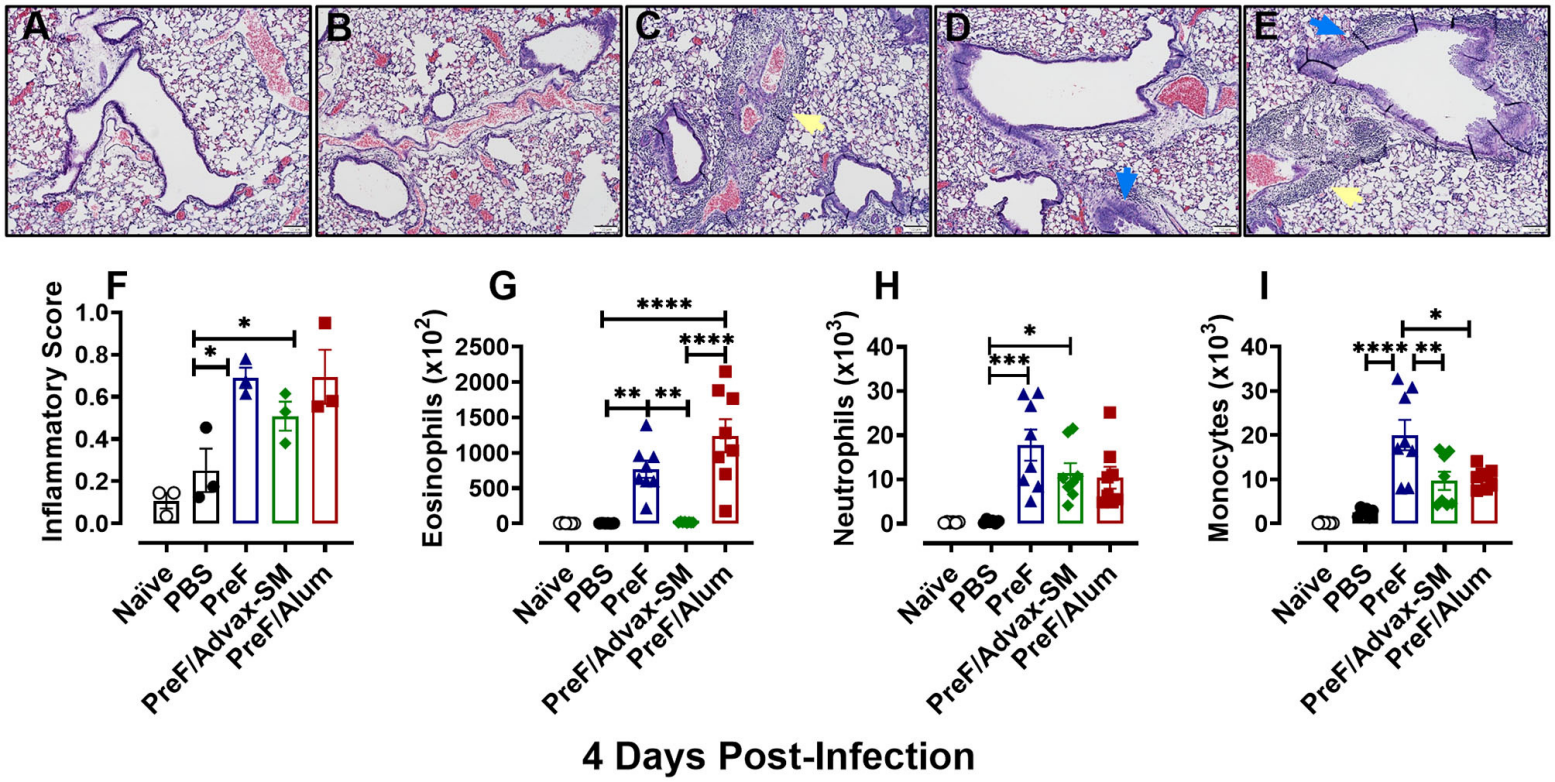
PreF and PreF/Alum immunization elicited enhanced pulmonary inflammation characterized by robust airway eosinophil recruitment. Naive mice were immunized and challenged with RSV as described in Figure 1. At 4 dpi, left lungs were formalin filled, paraffin embedded and sectioned for staining with H&E. Each panel represents an individual mouse from the indicated group (Scale bar 100 μm) **(A–E)**. Two blinded, independent pathologists scored all slides as described in the methods. Scores between the two investigators were averaged and data is represented as mean ± SEM (*n* = 3 mice) **(F)**. At 4 dpi, BAL was collected and eosinophils **(G)**, neutrophils **(H)**, and monocytes **(I)** were identified via flow cytometry. Data are represented as mean ± SEM (*n* = 6–8 mice per group);*p < 0.05, **p < 0.01, ***p < 0.001, and ****p < 0.0001. Yellow arrows highlight perivascular inflammation and blue arrows highlight peribronchial inflammation.

To evaluate potential immunopathology in mice that received Th2-skewing immunizations (PreF alone and PreF/Alum) as compared to the greater Th1-biased regimen (PreF/Advax-SM), left lungs were harvested from immunized mice at 4 dpi and stained with H&E to evaluate inflammation. Representative images (10X) taken from animals in each immunization group demonstrated increased inflammation in all immunization groups relative to PBS controls, with pronounced perivascular inflammation seen in PreF- and PreF/Alum-immunized animals (Figures 2A–E). Inflammatory scores increased at 4 dpi in all groups that received PreF-formulated immunizations but only PreF alone and PreF/Alum elicited significantly greater inflammation compared to PBS (Figure 2F). To determine the contribution of innate cellular responses to the enhanced inflammation observed in PreF- and PreF/Alum-immunized groups, bronchoalveolar lavage (BAL) was collected at 4 dpi and analyzed via flow cytometry (Gating strategy for discriminating innate immune cells is shown in Supplementary Figure 1). Immunization with PreF alone and PreF/Alum elicited a dramatic recruitment of eosinophils to the airways following RSV challenge, whereas eosinophil populations remained at baseline in PreF/Advax-SM-immunized mice (Figure 2G). Neutrophils were increased in the BAL of PreF- and PreF/Advax-SM-immunized mice compared to PBS controls (Figure 2H) and total monocytes were greater in PreF-immunized animals as compared to all other groups tested (Figure 2I). Taken together these results show that increased airway eosinophils paralleled increases in inflammation scores in mice immunized with PreF alone and PreF/Alum, whereas neutrophils and monocytes were more pronounced in the unadjuvanted PreF group.

### Increased Mucus Production Parallels Enhanced Inflammation in Mice Immunized With PreF Alone or PreF/Alum

**Figure 3:**
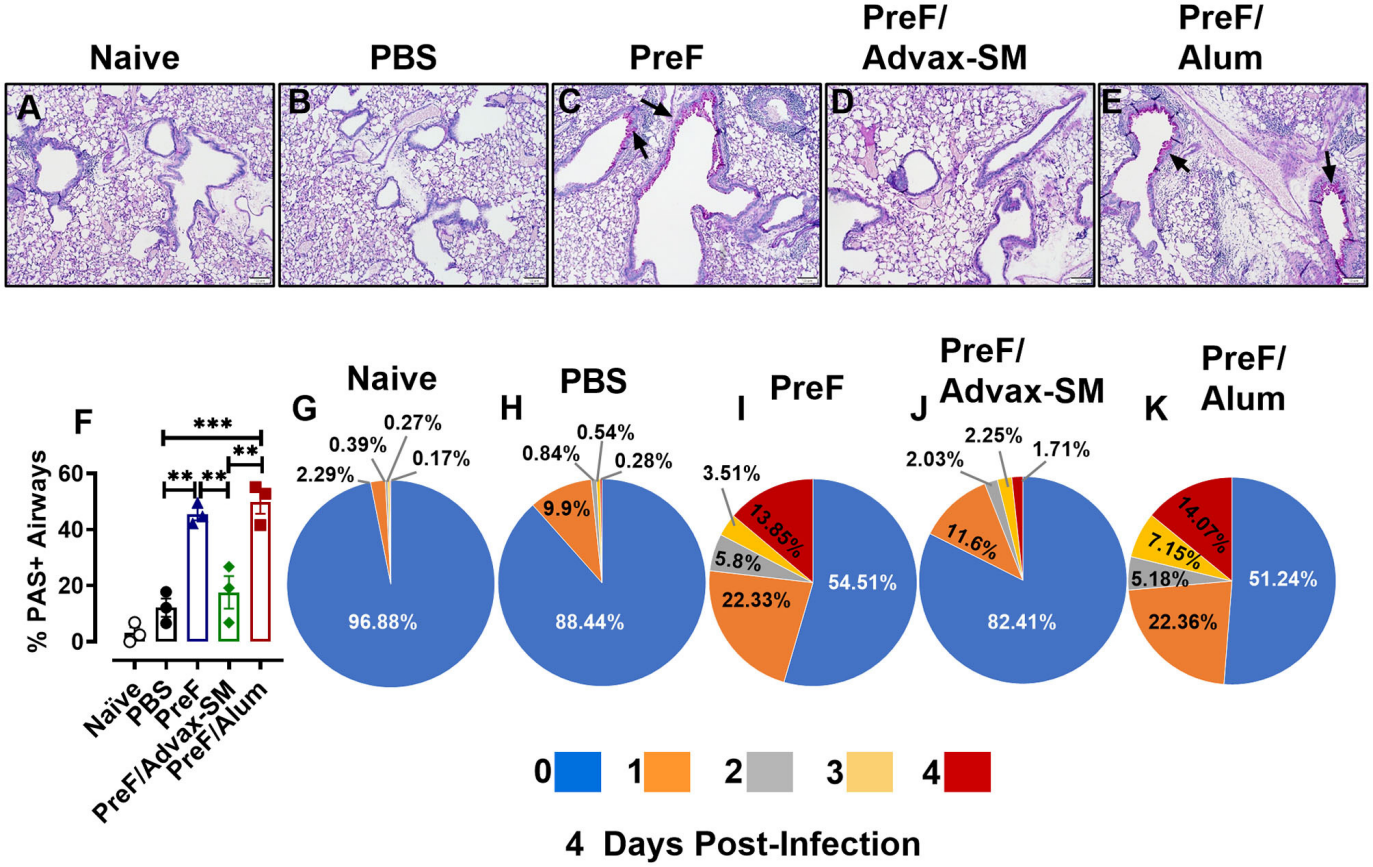
Increased mucus production parallels enhanced inflammation in mice immunized with PreF alone or PreF/Alum. Mice were immunized and challenged with RSV as described in Figure 1. At 4 dpi, lungs were formalin filled, paraffin embedded and sectioned for staining with PAS. Each panel represents an individual mouse from the indicated group (Scale bar 100 μm) **(A–E)**. To quantify the extent of PAS staining, lungs were scored as previously described in the methods. Scores were averaged and the total percentage of PAS+ airways were graphed (*n* = 3) **(F)**; **p < 0.01 and ***p < 0.001. A more detailed breakdown of scores for each cohort is provided, calculated as a proportion i.e., number of airways of each severity score (0–4) divided by the total number of airways, according to the methods **(G–K)**.

Human RSV disease is characterized by both inflammation and extensive mucus production. Thus, to determine if the enhanced inflammation seen in PreF- and PreF/Alum-immunized groups was also associated with enhanced mucus production, left lungs were harvested at 4 dpi and Periodic acid-Schiff (PAS) stained to analyze mucus metaplasia. Representative images (10X) were taken from a sample within each immunization group to visualize the extent of mucus production (Figures 3A–E). A majority of airways from naïve (Figure 3A), PBS (Figure 3B), and PreF/Advax-SM immunization groups (Figure 3D) had little to no PAS+ staining, while PreF alone (Figure 3C) and PreF/Alum (Figure 3E) groups had extensive PAS+ staining. Consistent with the representative lung sections, PreF– and PreF/Alum-immunization groups had higher percentages of PAS+ airways compared to all other groups tested (Figure 3F). To discriminate the degree of airway mucus production, the level of severity was reported for each group, whereby no PAS+ staining in the airway yielded a score of “0,” the frequency of airways with 1–25% PAS+ staining received a score of “1,” 26–50% PAS+ yielded a score of “2,” 51–75% PAS+ airways received a score of “3,” and 76–100% PAS+ staining received a score of “4.” Four days after diluent (Figure 3G) or RSV challenge, PBS (Figure 3H) and PreF/Advax-SM (Figure 3J) immunization groups had the largest percentage of unaffected airways and a small proportion of airways with mild PAS+ staining (1 score). In contrast, PreF- (Figure 3I) and PreF/Alum-immunized mice (Figure 3K) had smaller percentages of unaffected airways and greater frequency of airways with higher severity scores (2–4 scores).

### PreF/Advax-SM Immunization Produced Th1-Dominant Immunity With Increased RSV F-Specific CD8+ T Cells

**Figure 4:**
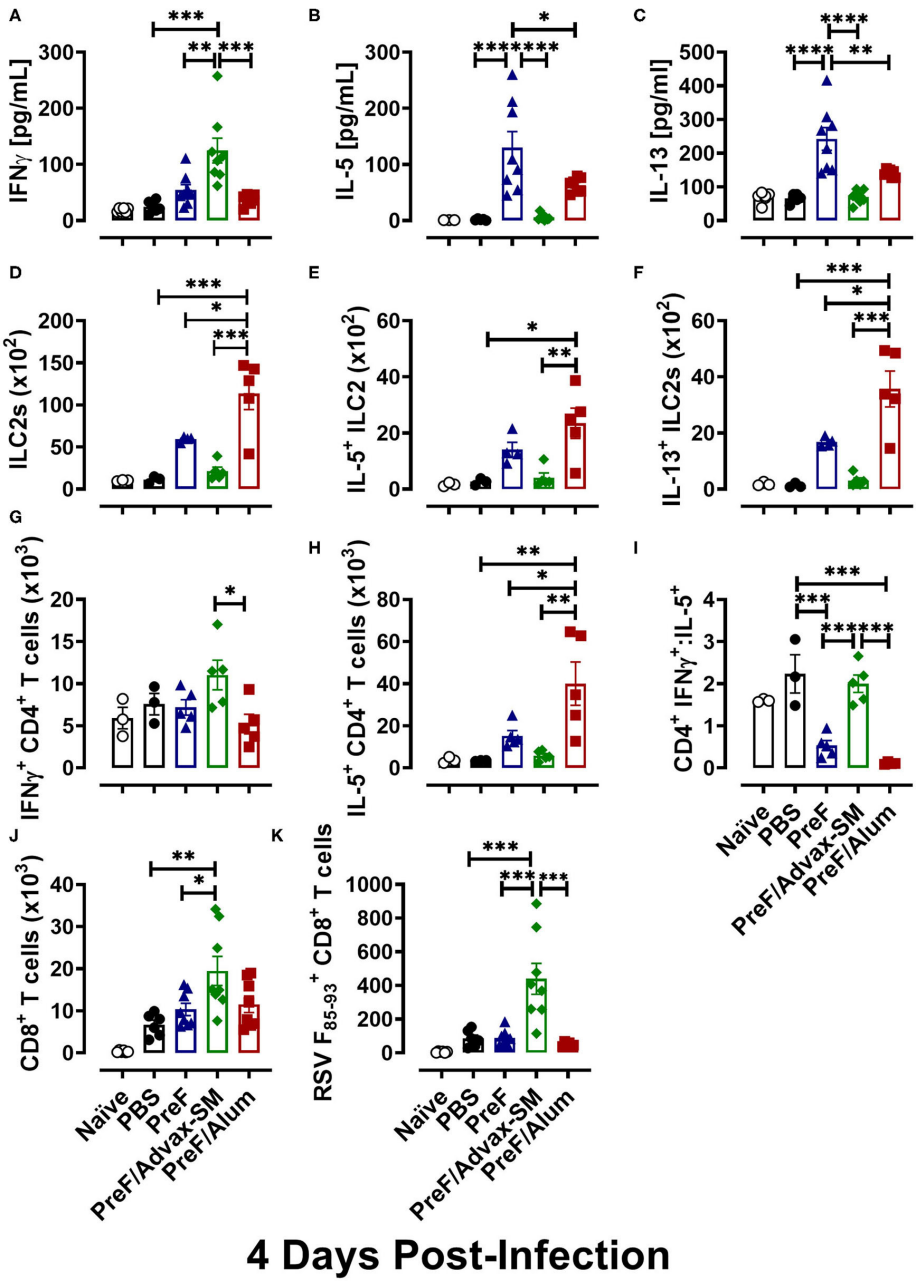
PreF/Advax-SM immunization produced Th1-dominant immunity with increased RSV F-specific CD8+ T cells. At 4 dpi, first wash samples were harvested from the airways for quantification of IFNγ **(A)**, IL-5 **(B)**, and IL-13 **(C)** by Luminex. Right lungs were harvested and homogenized for quantification of ILC2s **(D)** and ILC2 intracellular cytokine staining of IL-5 **(E)** and IL-13 **(F)** by flow cytometry. Intracellular production of IFNγ **(G)** and IL-5 **(H)** by CD4+ T cells from lung homogenate were quantified by flow cytometry and the ratio of IFNγ/IL-5-producing CD4+ T cells was calculated **(I)**. Total CD8+ T cells **(J)** and RSV F_85−93_-specific CD8+ T cells **(K)** were measured from BAL. Data are represented as mean ± SEM (*n* = 6–8 mice per group);*p < 0.05, **p < 0.01, ***p < 0.001, and ****p < 0.0001.

To delineate the relationship between distinct Th cell subsets and associated pulmonary inflammation and mucus production, Th1- and Th2-type cytokines were quantified from BAL. IFNγ, the canonical Th1 cytokine, was highest in PreF/Advax-SM mice at 4 dpi (Figure 4A), whereas levels of the Th2-associated cytokines, IL-5 (Figure 4B) and IL-13 (Figure 4C), were similar between PreF-Advax-SM-immunized mice and control groups (naïve and PBS). Conversely, PreF- and PreF/Alum-immunized mice had increased concentrations of IL-5 and IL-13 with no appreciable increase in IFNγ levels in these groups.

To identify cellular sources contributing to the Th1-associated cytokine bias of PreF/Advax-SM and the Th2-associated cytokine bias of PreF and PreF/Alum immunization, ILC2 and CD4+ T cell populations were analyzed from lung homogenate at 4 dpi via flow cytometry (Gating strategy to discriminate T cell populations and ILC2s are shown in Supplementary Figures 2, 3, respectively). Consistent with their Th2 cytokine profiles, PreF/Alum immunization elicited greater ILC2 populations (Figure 4D) following RSV challenge compared to all other groups. Though ILC2s trended higher in PreF alone immunization than control and PreF/Advax-SM groups, the difference was not significant. Moreover, PreF/Alum-immunized mice had increased IL5+ (Figure 4E) and IL-13+ (Figure 4F) ILC2 populations as compared to PreF/Advax-SM-immunized mice and PBS controls; once again, increased trends in the PreF alone group did not achieve significance. In conjunction with increased ILC2s, PreF/Alum-immunized mice had marked increases in IL-5-producing CD4+ T cells in lung homogenate (Figure 4H). PreF-immunized mice had similar trends in increased IL-5+ CD4+ T cells but were not significantly different compared to PBS controls. In contrast, PreF/Advax-SM-immunized mice had the largest population of IFNγ+ CD4+ T cells (Figure 4G) with distinctly increased IFNγ+:IL-5+ CD4+ T cell ratios (Figure 4I) as compared to PreF- and PreF/Alum-immunized mice. A similar increase in the ratio of IFNγ+:IL-5+ CD4+ T cells was observed in the PBS group.

Lastly, due to the importance of CD8+ T cells in clearing RSV, general CD8+ T cell populations and RSV F_85−93_-specific CD8+ T cells were compared in mice from each immunization group. Mice immunized with PreF/Advax-SM had increased total CD8+ T cells as compared to PreF-immunized mice and PBS controls (Figure 4J). Moreover, only PreF/Advax-SM-immunized mice had increased RSV F_85−93_-specific CD8+ T cells, which were greater than all other groups tested (Figure 4K). Collectively, these results identified ILC2s and CD4+ T cells as cellular sources of Th2-type cytokines associated with PreF- and PreF/Alum-immunized mice following RSV challenge. In stark contrast, PreF/Advax-SM immunization elicited an increase in IFNγ-producing CD4+ T cells and RSV F_85−93_-specific CD8+ T cells in response to RSV exposure that likely contributed to viral protection.

### Inflammation Worsens Over Time in Unimmunized Compared to Immunized Groups

**Figure 5:**
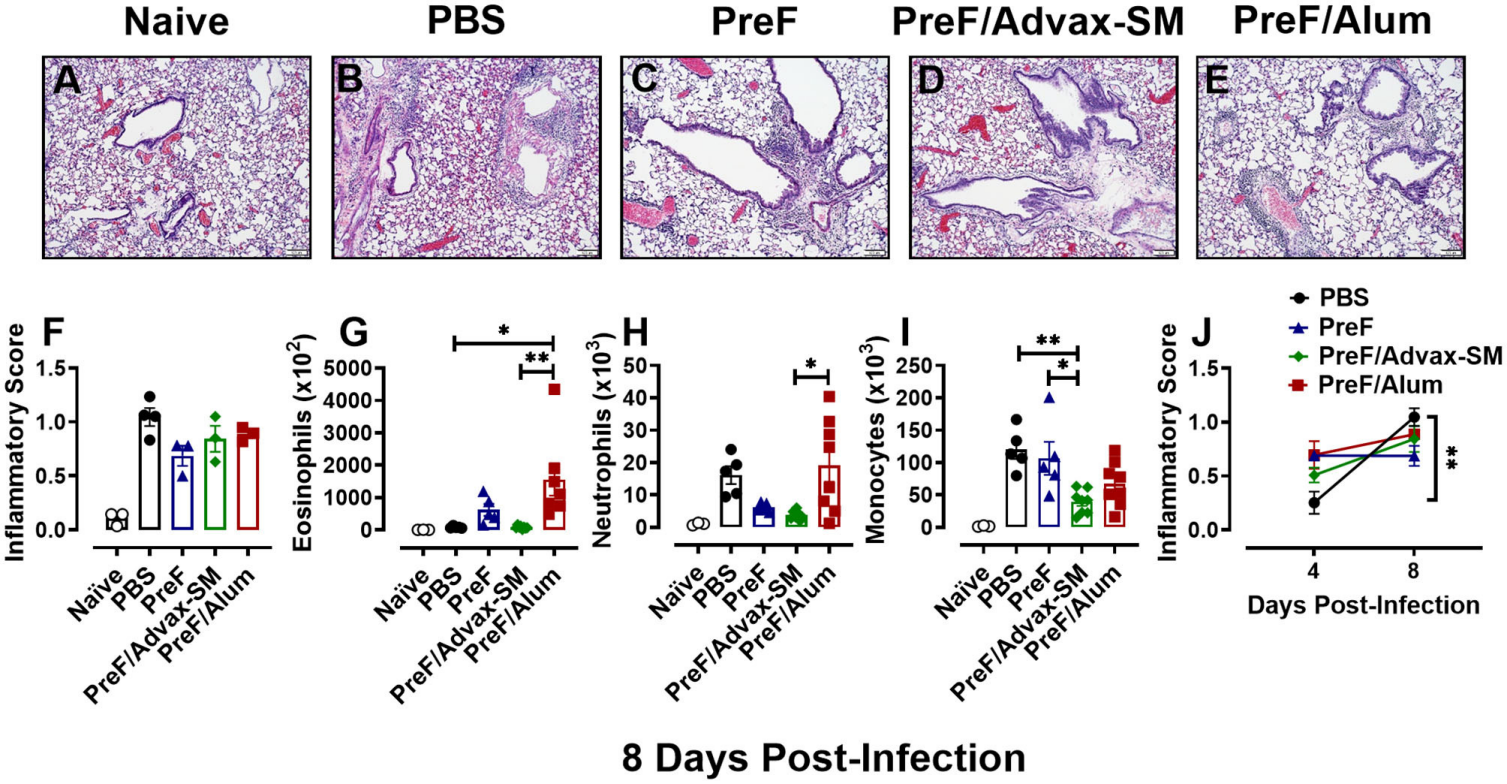
Inflammation worsens over time in unimmunized compared to immunized groups. At 8 dpi, left lungs were formalin fixed and stained with H&E to assess inflammation. Each panel represents an individual mouse from the indicated group (Scale bar 100 μm) **(A–E)**. Two blinded, independent pathologists scored all slides and scores were averaged. Data is represented as mean ± SEM (*n* = 3–4 mice) **(F)**. BAL was collected at 8 dpi for quantification of eosinophils **(G)**, neutrophils **(H)**, and monocytes **(I)** via flow cytometry. Data are represented as mean ± SEM (*n* = 6–8 mice). Inflammatory scores from 4 and 8 dpi were compared for each immunization groups over time **(J)**. Data are represented as mean ± SEM (*n* = 3–4 mice); *p < 0.05 and **p < 0.01.

To determine if immunization with PreF, PreF/Alum, or PreF/Advax-SM expedites disease resolution, innate inflammatory cells and lung inflammation were examined later at 8 days post-RSV challenge. Representative images (10X) were taken from H&E stained lung sections from each immunization group (Figures 5A–E). Each RSV-challenged group displayed enhanced inflammation that was predominately perivascular in nature with non-significant reductions in inflammation in the immunization groups compared to the PBS group (Figure 5F). Similar to the 4-day time point, PreF/Alum mice maintained greater airway eosinophil populations (Figure 5G) as compared to PreF/Advax-SM-immunized mice and PBS controls. Neutrophils were elevated in PBS controls and the PreF/Alum group compared to PreF/Advax-SM-immunized mice, although no significant difference was observed in the PBS group (Figure 5H). PreF-immunized and PBS control groups had the greatest number of monocytes in the BAL when compared to PreF/Advax-SM-immunized mice (Figure 5I). Overall, populations of innate inflammatory cells had largely resolved in the BAL of PreF/Advax-SM-immunized mice by 8 dpi. In PBS controls, increases in neutrophils and monocytes likely contributed to the dramatic increase in inflammation between 4 and 8 dpi, whereas inflammatory scores for the groups immunized with PreF remained largely unchanged (Figure 5J). These results suggest that inflammation and recruitment of inflammatory cells dramatically increased in PBS controls, innate inflammatory cells largely resolved in the PreF/Advax-SM group, and PreF/Alum immunization failed to resolve airway eosinophilia by 8 days post-RSV challenge.

### Mucus Production Persisted in the Lungs of PreF/Alum-Immunized Mice at 8 dpi and Remained Low in Mice Immunized With PreF/Advax-SM

**Figure 6:**
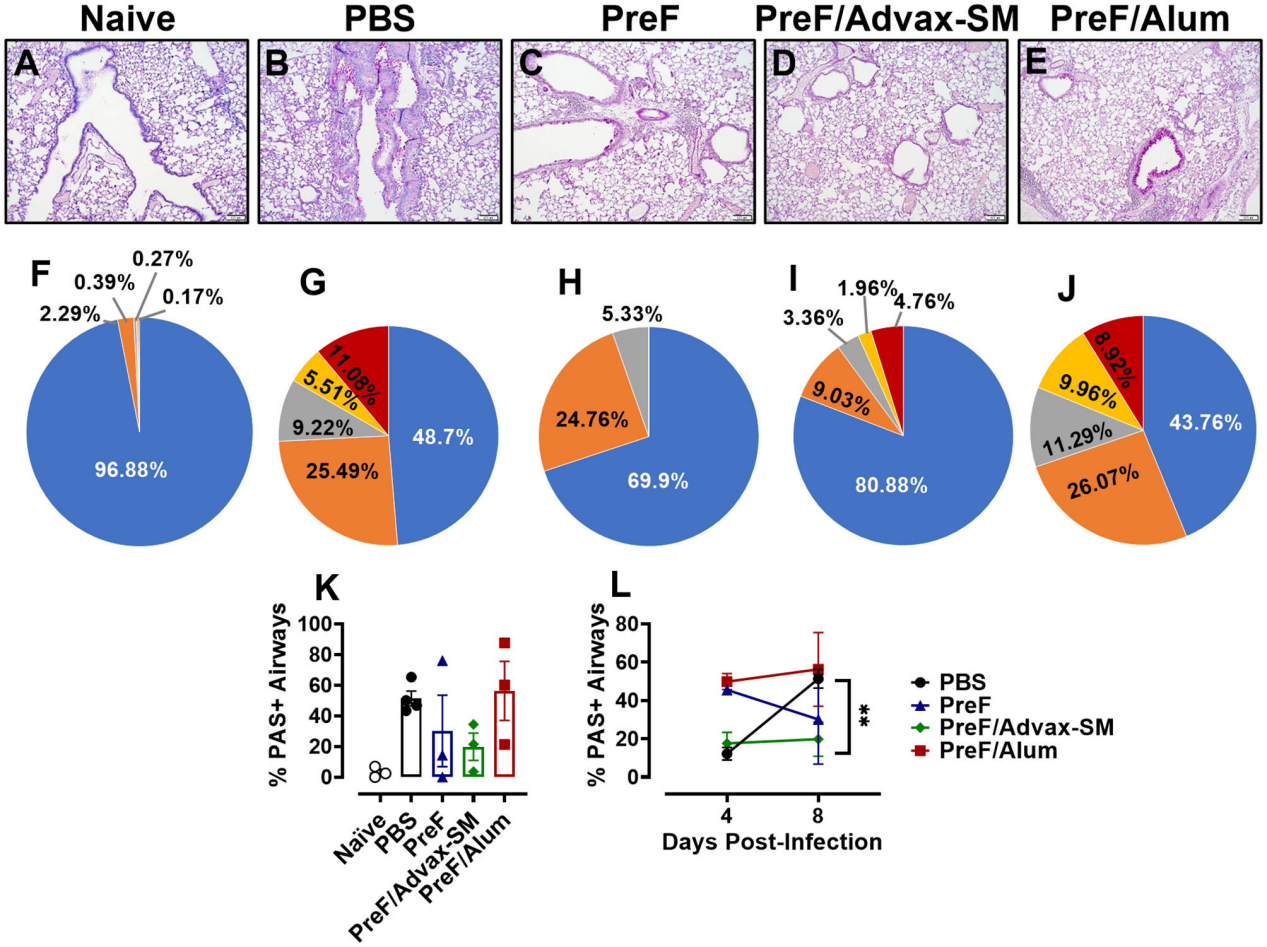
Mucus production persisted in the lungs of PreF/Alum-immunized mice at 8 dpi and remained low in mice immunized with PreF/Advax-SM. At 8 dpi, left lungs were formalin fixed and stained with PAS to assess mucus production. Each panel represents an individual mouse from the indicated group (Scale bar 100 μm) **(A–E)**. A more detailed breakdown of scores for each cohort is provided, calculated as a proportion i.e., number of airways of each severity score (0–4) divided by the total number of airways, according to the methods **(F–J)**. To quantify the extent of PAS staining, lungs were scored as previously described in the methods. Scores were averaged and the total percentage of PAS+ airways were graphed. Total percentage PAS+ airways from 8 dpi are shown in **(K)** (*n* = 3–4) and are further compared over time between 4 and 8 dpi in each group in **(L)**; **p < 0.01.

To determine if mucus production resolved or worsened in immunized mice over the course of infection, left lungs were collected and PAS-stained at 8 dpi. Representative images (10X) were taken from samples in each group (Figure 6A–E). Readily discernable mucus was observed in PBS (Figure 6B) and PreF/Alum (Figure 6E) lung sections, with a lower frequency of PAS+ staining seen in mice immunized with PreF-alone (Figure 6C). Quantitative analysis of the percentage of PAS+ airways revealed that PBS controls and PreF/Alum-immunized groups had the highest proportion of PAS+ airways (Figure 6K). PreF/Advax-SM immunized mice maintained a low percentage of PAS+ airways through 8 dpi. A more detailed analysis revealed that >50% of airways were PAS+ in PBS– (Figure 6G) and PreF/Alum-immunized mice (Figure 6J) and these groups had the largest proportions of airways that received higher severity scores (scores of 2–4) relative to all other groups (Figure 6F–J). At 8 dpi, the majority of airways in the PreF-immunization group were unaffected and when PAS+ was observed, it was generally mild (score of 1; Figure 6I). Mice immunized with PreF/Advax-SM (Figure 6J) had the largest percentage of PAS-free airways, with a smaller proportion of airways receiving higher severity scoring (scores of 2–4). Similar to what was seen with inflammation over time, PBS controls demonstrated a distinct enhancement of mucus production between 4 and 8 dpi (Figure 6L), while PreF/Alum immunization elicited a high degree of PAS+ staining throughout the RSV time course. In contrast, by 8 dpi, PreF-immunized mice resolved some of the high proportion of PAS+ airways observed at 4 dpi, while mice immunized with PreF/Advax-SM maintained low levels of mucus production. Together, these data suggest that the high degree of mucus produced by 8 dpi in PBS controls is mitigated more effectively in mice immunized with PreF/Advax-SM as opposed to PreF/Alum immunization.

### Th2-Dominant Immunity of PreF/Alum and the Th1-Dominant Immunity of PreF/Advax-SM Persisted Through 8 dpi

**Figure 7:**
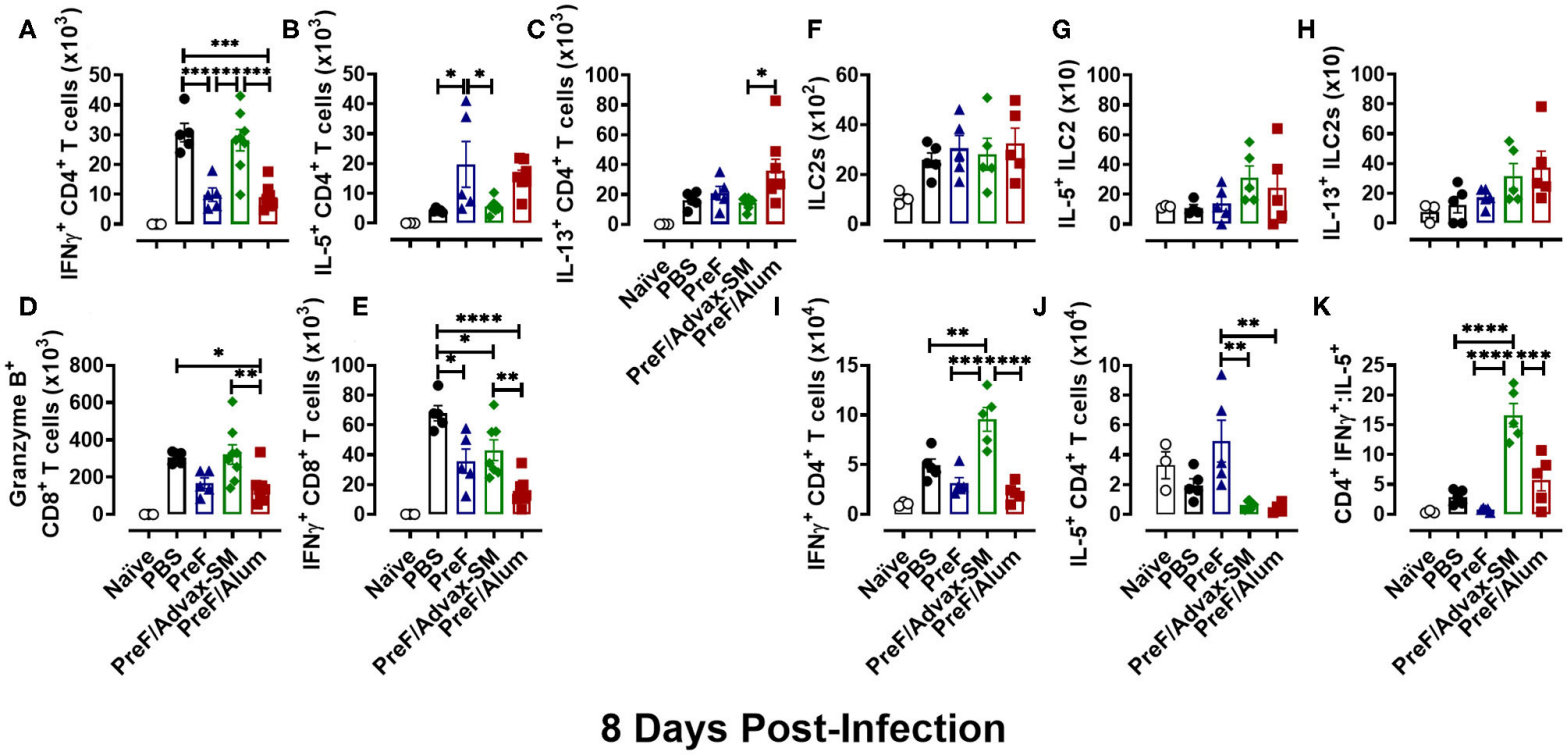
Th2-dominant immunity of PreF/Alum and the Th1-dominant immune responses of PreF/Advax-SM persisted through 8 dpi. At 8 dpi, BAL samples collected for analysis of IFNγ+ **(A)**, IL-5+ **(B)**, and IL-13+ **(C)** CD4+ T cells, and Granzyme B+ **(D)** and IFNγ+ **(E)** CD8+ T cells via flow cytometry. Right lungs were also harvested at 8 dpi and processed for analysis of ILC2 populations **(F)**, IL-5+ ILC2s **(G)**, and IL-13+ ILC2s **(H)**, as well as, IFNγ+ **(I)**, IL-5+ **(J)** CD4+ T cells by flow cytometry; the ratio between IFNγ+/IL-5+ CD4+ T cells was also calculated **(K)** (*n* = 3–5); *p < 0.05, **p < 0.01, ***p < 0.001, and ****p < 0.0001.

Based on the changes in lung pathology observed between immunization groups at 8 dpi, we asked whether there was an associated change in Th phenotypes among immunization groups at 8 dpi (Th1 in PreF/Advax-SM; Th2 in PreF alone and PreF/Alum). Analysis of T cell responses in the BAL showed the highest number of IFNγ+ CD4+ T cells in PBS- and PreF/Advax-SM-immunized mice (Figure 7A), while mice immunized with PreF alone and PreF/Alum had larger populations of IL-5+ CD4+ T cells (Figure 7B). Additionally, IL-13+ CD4+ T cell populations in PreF/Alum immunized mice were significantly higher than PreF/Advax-SM animals (Figure 7C). Examination of CD8+ T cell populations revealed greater Granzyme B+ (Figure 7D) and IFNγ+ CD8+ T cells + (Figure 7E) in the airways of PreF/Advax-SM immunized mice and PBS controls, with PBS mice having significantly more IFNγ+ CD8+ T cells than the other immunization groups tested.

In addition to cellular analysis in the BAL, T cells and ILC2s were measured in lung homogenate to address potential differences in cell localization. In accordance with their role as early immune responders, ILC2 populations (Figure 7F) had contracted by 8 dpi and no differences were detected between immunization groups. Moreover, populations of IL-5+- (Figure 7G) and IL-13+-producing ILC2s (Figure 7H) were similar between all groups. Finally, Th phenotypes were examined at 8 dpi in lung homogenate. PreF/Advax-SM immunized mice had the highest number of IFNγ+ CD4+ T cells (Figure 7I) compared to other groups, while IL-5+ CD4+ T cells (Figure 7J) were greatest in PreF-vaccinated mice. Consistent with the data at 4 dpi, the CD4+ IFNγ+:IL-5+ ratio (Figure 7K) was higher in PreF/Advax-SM immunized mice as compared to all other groups tested. This demonstrates the persistent Th1-dominant immune response elicited by PreF/Advax-SM immunization.

## Discussion

Here, we explored the ability of a RSV preF subunit protein-based immunization to protect naïve BALB/c mice from RSV infection and pulmonary pathology when formulated with either a Th2- or more Th1/Th2-balanced adjuvant. Alum has been in use in human vaccines since the early 20th century and is well recognized for its Th2-skewing and antibody boosting properties. As expected, PreF/Alum immunization produced Th2-biased immunity with high titers of PreF-specific IgG1 and neutralizing antibody, which were associated with undetectable viral replication following RSV challenge. While PreF alone lacked the immunogenicity to offer complete RSV protection, likely due to low neutralizing antibody production, PreF-immunized mice generated Th2-type immune responses similar to mice immunized with PreF/Alum. On the other hand, Advax-SM is a delta-inulin polysaccharide adjuvant formulated with CpG55.2 ODN, a TLR9 agonist. The combination of delta inulin and TLR9 agonism has been reported to generate a Th1/Th2-balanced adjuvant response in models of SARS-associated coronavirus, Japanese encephalitis virus, and West Nile virus (24, 31, 32). In models of RSV immunization, TLR9 agonists formulated with formalin-inactivated RSV have increased immunogenicity while ameliorating pulmonary pathology and reducing airway hyperresponsiveness normally exacerbated by FI-RSV immunization (33, 34). In one report, an intramuscular immunization of live RSV formulated with Advax-SM protected from RSV infection with increased neutralizing antibody titers and greater RSV-specific IgG2a/IgG1, but had similar lung inflammation as unadjuvanted control mice, suggesting the Th2 polarization of the live RSV vaccine may have overcome the greater Th1 bias of the CpG oligonucleotide (23). These studies support the idea that TLR9 agonists may be combined with stabilized PreF proteins to induce a more Th1-biased response with an acceptable safety profile and highlight the importance of evaluating adjuvant safety as well as efficacy when developing vaccine strategies.

In our study, mice immunized with PreF/Advax-SM elicited a greater Th1-type response, while still generating high levels of PreF-specific IgG2a and IgG1 antibody. Despite producing lower neutralizing antibody titers than PreF/Alum immunized, PreF/Advax-SM mice were resistant to RSV infection. Moreover, the discreet Th profiles produced by PreF/Alum and PreF/Advax-SM generated distinct differences in pulmonary pathology. The Th2 profile of PreF/Alum-immunized mice was associated with enhanced pulmonary inflammation and mucus production at 4 dpi and continued to have the greatest proportion of PAS+ airways as late as 8 dpi. The immunopathology associated with PreF/Alum immunization occurred in spite of high neutralizing antibody titers and undetectable viral replication. Previous studies of FI-RSV-associated ERD have demonstrated that poor avidity and affinity maturation resulted in non-protective antibody development and Th2-associated immunopathology (19). Furthermore, it has been suggested that alum did not alter the ERD profile induced by FI-RSV immunization (18). However, in our study, alum adjuvant exacerbated the pathology induced by immunization with PreF alone despite protection from RSV infection. In contrast, the Th1-type responses generated by immunization with PreF/Advax-SM elicited less overall inflammation with low levels of mucus production. Importantly, PreF/Advax-SM immunization protected mice from the worsening inflammation and mucus production that occurred between 4 and 8 dpi in PBS controls. Taken together, these data indicate that RSV PreF immunization formulated with Th1-skewing adjuvants, like Advax-SM, provide protection against RSV infection in naïve BALB/c mice as compared to alum by inhibiting viral replication without eliciting enhanced pulmonary pathology. Though IgE was not measured in these studies, Alum, as opposed to CpG-ODN, adjuvanted antigens have been associated with an increased production of IgE (35). Future studies will be needed to address the role of IgE following immunization with RSV PreF adjuvanted with Alum.

Neutralizing antibodies have been shown to reduce the severity of RSV disease (12, 13). Moreover, the risk of re-infection is inversely correlated to the level of serum neutralizing antibodies (36). As such, boosting serum neutralizing antibody titers has been an important objective of RSV vaccination, especially since the discovery and subsequent stabilization of the pre-fusion conformation of RSV F protein (11). RSV-neutralizing activity in human sera is primarily derived from PreF-specific antibodies (37, 38). Thus, the use of stabilized PreF as a vaccine antigen has sparked new hope that high neutralizing antibody titers can be produced to provide long-term protection. Pre-clinical studies in young and aged mice have demonstrated the neutralizing antibody boosting-potential of RSV PreF when formulated with both Th2- and Th1/Th2-skewing adjuvants (39). In agreement with our data, PreF immunization alone has been shown to lack sufficient immunogenicity to increase neutralizing antibody titers above a protective threshold (39). Our study expanded these findings by directly challenging PreF-immunized mice and demonstrated incomplete protection and enhanced lung pathology following RSV infection in association with little to no measurable neutralizing antibody production (i.e., only 50% of PreF-immunized mice produced any measurable neutralizing antibody). Earlier work found that higher titers of neutralizing antibody were associated with RSV PreF immunization formulated with Th1/Th2-balanced adjuvants (39). In contrast, our results show that the Th2-dominant immune response of PreF/Alum immunization produced higher neutralizing antibody titers as compared to the Th1-biased immune response elicited by PreF/Advax-SM immunization. Importantly, however, both PreF/Alum and PreF/Advax-SM immunization fully protected mice from RSV infection with a complete absence of detectable viral replication in the lungs at 4 dpi. Additional studies will be required to determine if lower neutralizing antibody production, as seen in PreF/Advax-SM, affects the durability of protection against RSV challenge.

Despite evidence of the correlation between neutralizing antibody in the serum and protection from severe RSV disease (12, 13, 36, 37), other data suggests that high serum levels of neutralizing antibody provide insufficient protection from RSV-disease or reinfection in some individuals (40–42). Therefore, RSV vaccines that rely on neutralizing antibody as the sole correlate of protection against RSV disease may not provide universal or long-lasting immunity. Numerous studies have demonstrated the importance of T cells in clearing RSV in primary RSV infection (43–45). In mouse models of primary infection, CD8+ T cells are critical for viral control and both the number of CD8+ T cells and their production of IFNγ are crucial for effective clearance (46–48). Despite conferring protection against RSV replication, immunopathology has been linked to IFNγ-producing CD8+T cells during primary infection (47) and in memory responses resulting from immunization (49). In contrast, our data shows that immunization with PreF/Advax-SM protects against RSV infection and elicits RSV F-specific and IFNγ-producing CD8+ T cells without inducing additional pulmonary pathology. Alternatively, by 8 dpi, a robust IFNγ+ CD8+T cell response was induced in PBS-immunized control mice that was further associated with increased inflammation. These divergent outcomes in lung pathology associated with IFNγ+ CD8+T cells may be explained by the presence of pre-existing neutralizing antibody in PreF/Advax-SM as compared to the lack of pre-existing neutralizing antibody in PBS controls. Another model of RSV vaccination has demonstrated that pre-existing neutralizing antibody can protect mice from severe immunopathology caused by potent memory IFNγ-producing CD8+T cells (50). Therefore, RSV vaccination approaches that combine PreF with Th1/Th2-balanced adjuvants may prove to be efficacious and safe through the combination of increased neutralizing and RSV-specific antibody production and memory IFNγ+ CD8+T cell responses.

Like CD8+ T cells, research has demonstrated the importance of CD4+ T cells in the control of RSV infection and their role in inducing pathology (43–45). The 1960's vaccine trials of formalin-inactivated RSV formulated with alum (FI-RSV) demonstrated the sobering potential for vaccine-associated ERD. Upon natural RSV exposure, 80% of vaccinees developed ERD, requiring hospitalization and two children died (16). Investigations into the causes of ERD using multiple animal models have revealed Th2 CD4+ T cells to be closely linked to the increased inflammation, mucus, and airway hyperresponsiveness observed in ERD (51–55). In our study, both PreF alone and PreF/Alum immunization elicited Th2 CD4+ T cell profiles associated with enhanced inflammation, airway eosinophils, and extensive mucus production. Interestingly, despite their similar Th2 phenotypes, PreF-immunized mice resolved airway neutrophils, eosinophils, and mucus more rapidly than PreF/Alum immunized mice. This resolution occurred in PreF-immunized mice despite their poor neutralizing antibody production and detectable RSV replication, which was likely the cause of increased inflammatory innate cell recruitment as compared to PreF/Alum immunization. Whereas, mice immunized with PreF/Alum had high titers of neutralizing antibody and undetectable RSV in the lung, yet persistent airway mucus and inflammation. In contrast to data showing the ability of pre-existing antibody to temper the immunopathology induced by IFNγ+ CD8+T cells (50), our data suggests that pre-existing neutralizing antibody alone may not modulate the immunopathology associated with Th2 CD4+ T cells in a similar manner.

In addition to increases in Th2 CD4+ T cells, PreF- but especially PreF/Alum-immunization was associated with large populations of ILC2s producing IL-5 and IL-13 in the lung. ILC2s are the dominant innate lymphoid cell in the lung and are well recognized for their ability to produce large amounts of type 2 cytokines upon stimulation (56). In primary RSV, ILC2 expansion and activation result from damage to airway epithelial cells via RSV infection and subsequent release of IL-33 (57) and/or thymic stromal lymphopoietin (TSLP) (58). However, in PreF/Alum immunized animals, ILC2s increased and became activated in spite of effective RSV neutralization and undetectable virus in the lung at 4 dpi. Our data suggests that an alternative mechanism may contribute to the induction of ILC2 responses in PreF/Alum immunized mice. A murine model of *Nippostrongylus brasiliensis* demonstrated that crosstalk between ILC2s and antigen-specific Th2 CD4+ T cells occurs, promoting ILC2 proliferation and IL-13 production (59). This ILC2-CD4+ T cell crosstalk was shown to be mutually beneficial to both cell types' maintenance, expansion, and cytokine production. Future studies are necessary to understand how PreF/Alum immunization contributes to ILC2 expansion and activation. However, this is the first study to suggest a relationship between ILC2 activation and Th2-associated immunopathology following PreF/Alum immunization in naïve mice.

In conclusion, our studies demonstrated that PreF/Advax-SM and PreF/Alum immunizations provided equivalent protection from RSV infection but elicited dramatically different outcomes regarding lung pathology. The Th2 immunity induced by PreF/Alum immunization was associated with increased inflammation and abundant mucus production that persisted through 8 dpi. In addition to IL-5- and IL-13-producing CD4+ T cells, PreF/Alum immunization was also associated with increased IL-5- and IL-13-producing ILC2 populations. This previously unrecognized relationship between PreF/Alum-induced Th2 immunity and ILC2 activation in the absence of RSV replication may have important implications for RSV vaccine development. It suggests that despite eliciting high titers of neutralizing antibody, the extensive Th2 immune bias generated by PreF/Alum may still lead to overt pathology in naïve individuals. It has been suggested that RSV exposure prior to immunization may mitigate the overwhelming Th2 immunity and lung pathology observed here with PreF/Alum, however, considering the important safety concern, further studies are needed to confirm this hypothesis. In contrast to the Th2-skewed immunity of PreF/Alum, the combination of neutralizing antibody production, Th1 immunity, and cytotoxic IFNγ-producing CD8+T cells induced by PreF/Advax-SM provided vigorous protection from RSV replication without inducing pathology. Taken together, these data suggest that RSV PreF protein subunit vaccinations formulated with Th1/Th2-balanced adjuvants may provide complete RSV protection with a desirable safety profile. Future studies will be needed to elucidate safety and protection of preimmune mice when immunized with PreF alone or when formulated with a Th1- vs Th2-biased adjuvant.

## Data Availability Statement

The raw data supporting the conclusions of this article will be made available by the authors, without undue reservation.

## Ethics Statement

The animal study was reviewed and approved by The University of Pittsburgh Institutional Animal Care and Use Committee.

## Author Contributions

KEi, JK, and KEm contributed to the overall study design, execution, and interpretation of data and writing of the paper. SG and AZ contribute through development of novel assays for generation of data. ML contributed to data interpretation, figure development, and writing. MY contributed to data generation, study design, and analysis. TP and TO contributed to the analysis and interpretation of data. CM and NP contributed to study design. All authors contributed to the article and approved the submitted version.

## Conflict of Interest

NP is affiliated with Vaxine Pty Ltd., which hold commercial interests in Advax adjuvants CM and MY are affiliated with Calder Biosciences, which holds commercial interests in the stabilized PreF protein. The remaining authors declare that the research was conducted in the absence of any commercial or financial relationships that could be construed as a potential conflict of interest.
