# Supplementary material for: Prefusion RSV F Immunization Elicits Th2-Mediated Lung Pathology in Mice When Formulated With a Th2 (but Not a Th1/Th2-Balanced) Adjuvant Despite Complete Viral Protection
Source: Front Immunol. 2020 Jul 29;11:1673. doi: 10.3389/fimmu.2020.01673 (PMC7403488; doi:10.3389/fimmu.2020.01673)
Supplement: Supplementary file 1 [file Presentation_1.pptx]

## Slide 1
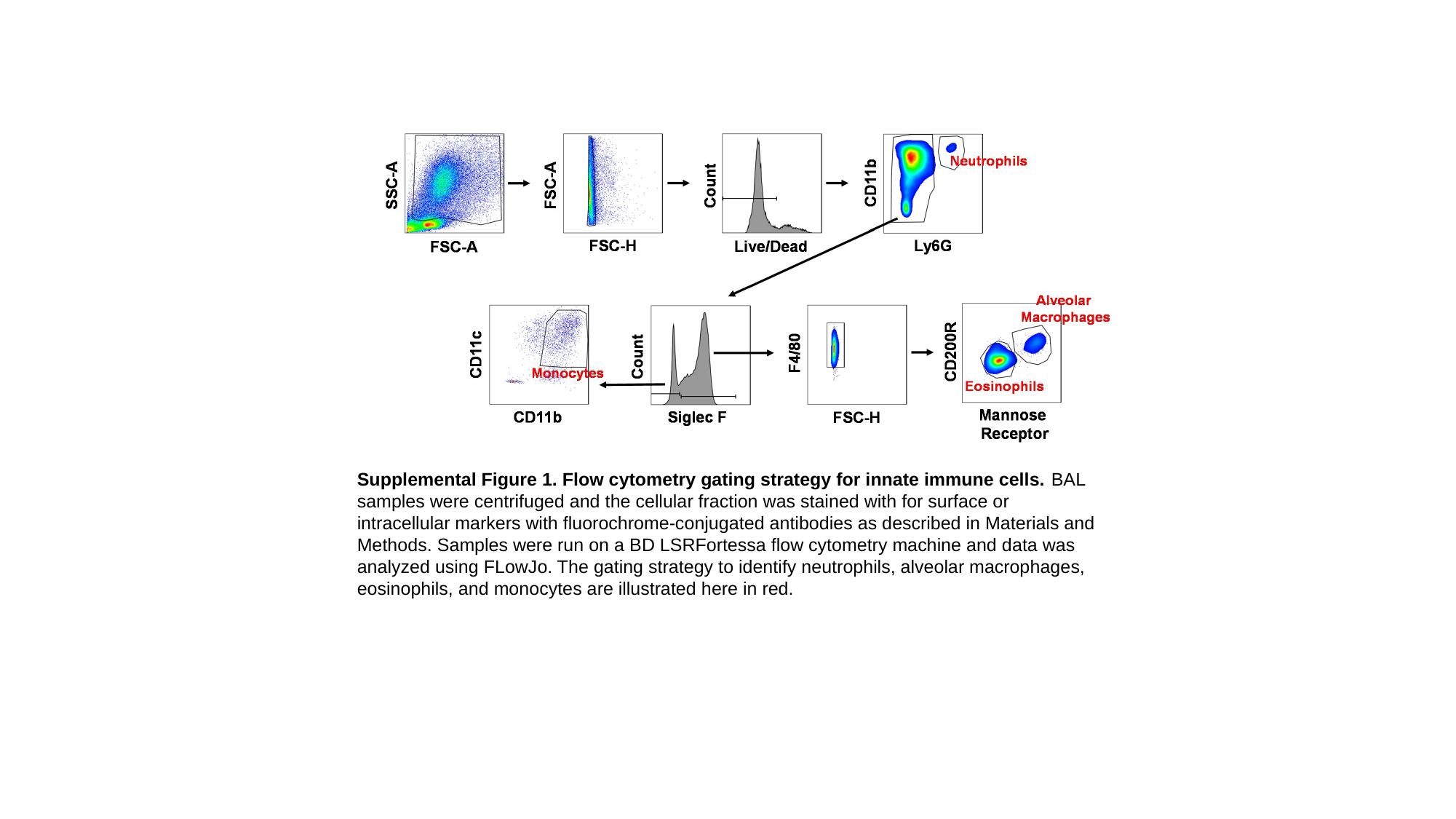

Supplemental Figure 1. Flow cytometry gating strategy for innate immune cells. BAL samples were centrifuged and the cellular fraction was stained with for surface or intracellular markers with fluorochrome-conjugated antibodies as described in Materials and Methods. Samples were run on a BD LSRFortessa flow cytometry machine and data was analyzed using FLowJo. The gating strategy to identify neutrophils, alveolar macrophages, eosinophils, and monocytes are illustrated here in red.

## Slide 2
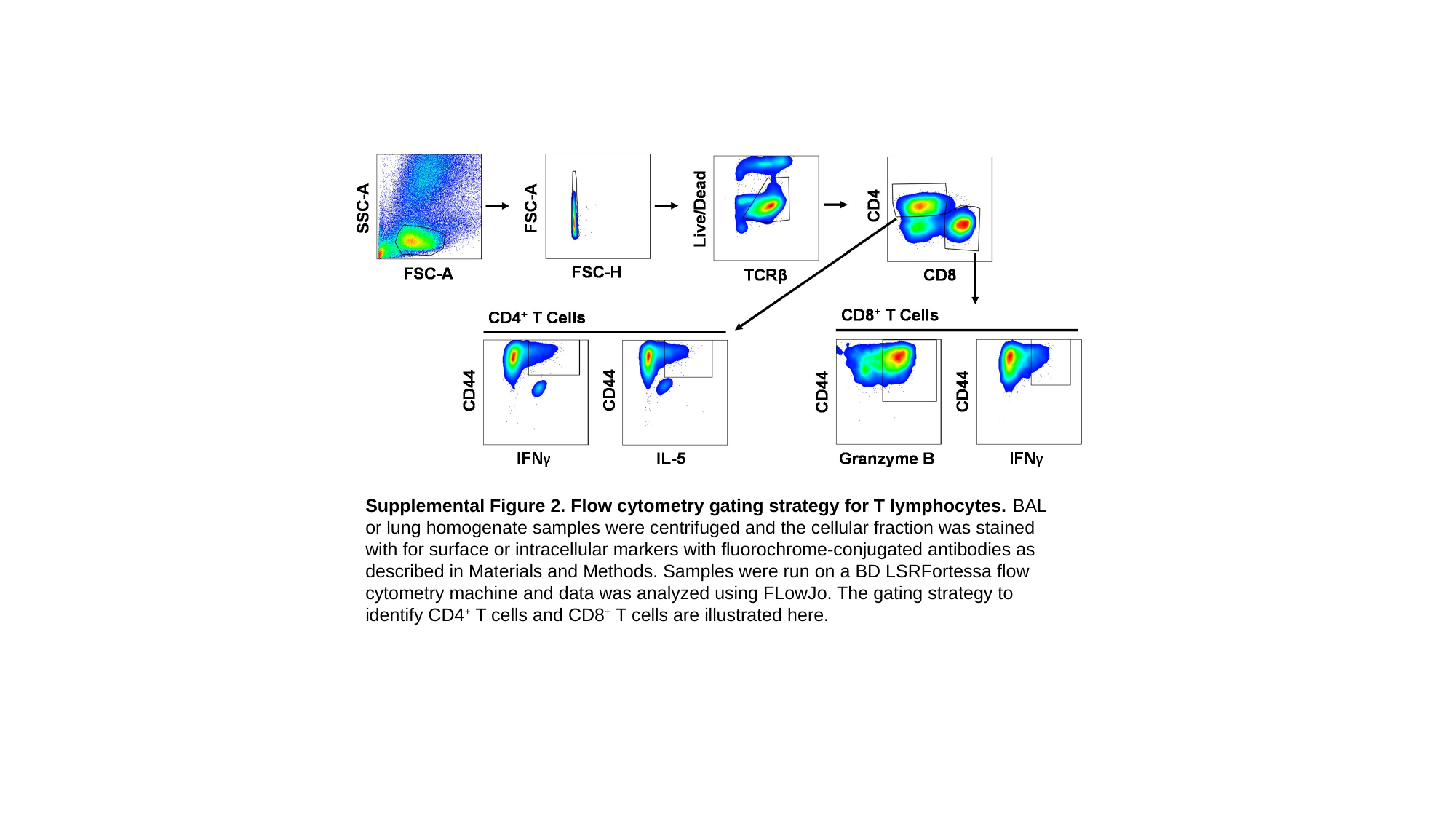

Supplemental Figure 2. Flow cytometry gating strategy for T lymphocytes. BAL or lung homogenate samples were centrifuged and the cellular fraction was stained with for surface or intracellular markers with fluorochrome-conjugated antibodies as described in Materials and Methods. Samples were run on a BD LSRFortessa flow cytometry machine and data was analyzed using FLowJo. The gating strategy to identify CD4+ T cells and CD8+ T cells are illustrated here.

## Slide 3
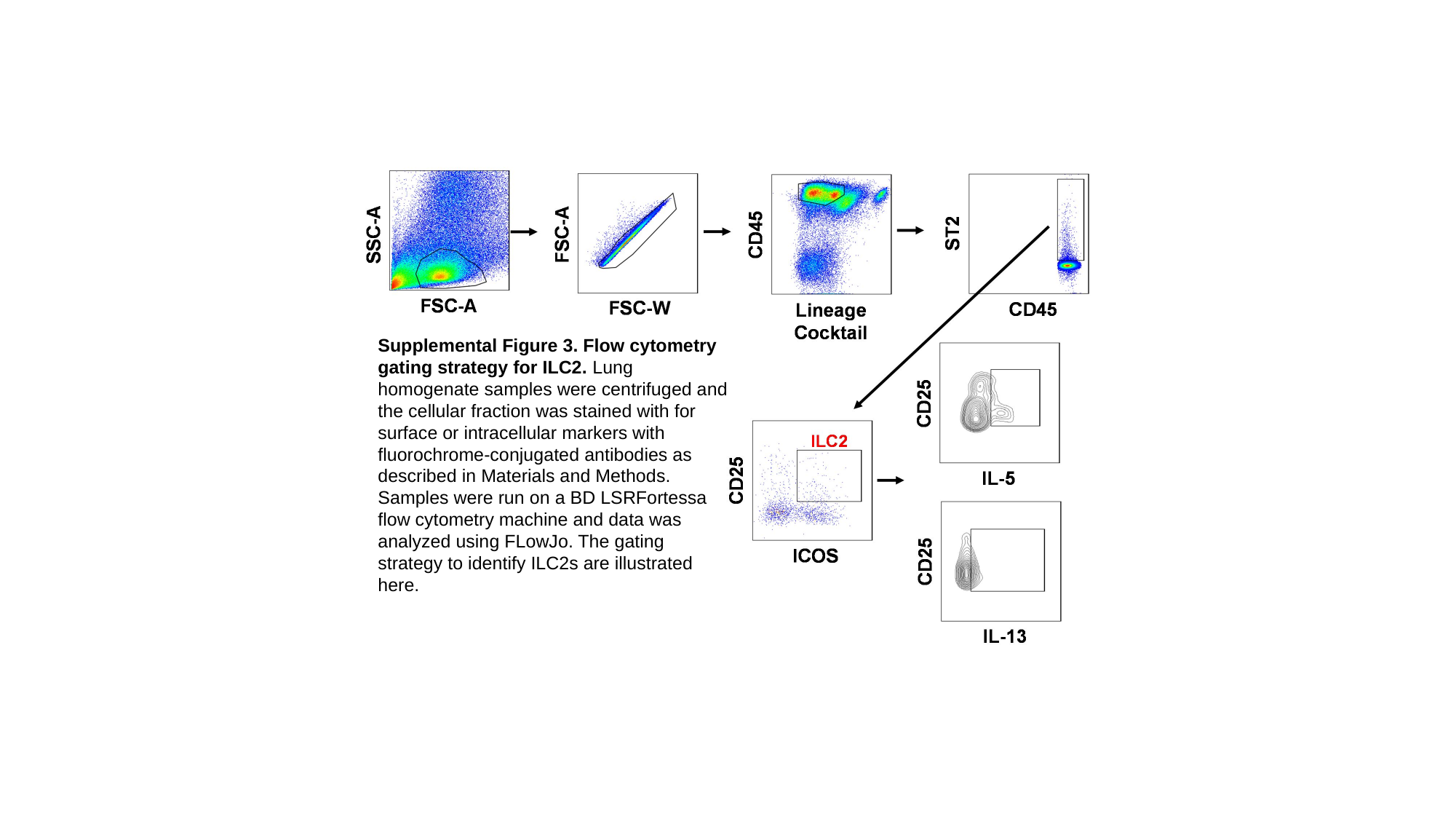

Supplemental Figure 3. Flow cytometry gating strategy for ILC2. Lung homogenate samples were centrifuged and the cellular fraction was stained with for surface or intracellular markers with fluorochrome-conjugated antibodies as described in Materials and Methods. Samples were run on a BD LSRFortessa flow cytometry machine and data was analyzed using FLowJo. The gating strategy to identify ILC2s are illustrated here.
